# Validation of MINORMIX Approach for Estimation of Low Birthweight Prevalence Using a Rural Nepal Dataset

**DOI:** 10.1093/jn/nxab417

**Published:** 2021-12-09

**Authors:** Karen T Chang, Emily D Carter, Luke C Mullany, Subarna K Khatry, Simon Cousens, Xiaoyi An, Julia Krasevec, Steven C LeClerq, Melinda K Munos, Joanne Katz

**Affiliations:** Department of International Health, Johns Hopkins Bloomberg School of Public Health,, Baltimore, MD, USA; Department of International Health, Johns Hopkins Bloomberg School of Public Health,, Baltimore, MD, USA; Department of International Health, Johns Hopkins Bloomberg School of Public Health,, Baltimore, MD, USA; Johns Hopkins University Applied Physics Laboratory, Laurel, MD, USA; Nepal Nutrition Intervention Project–Sarlahi, Lalitpur, Nepal; Maternal Adolescent Reproductive & Child Health (MARCH) Centre, London School of Hygiene and Tropical Medicine, London, UK; Data and Analytics Section, Division of Data, Analytics Planning and Monitoring, UNICEF, New York, NY, USA; Data and Analytics Section, Division of Data, Analytics Planning and Monitoring, UNICEF, New York, NY, USA; Department of International Health, Johns Hopkins Bloomberg School of Public Health,, Baltimore, MD, USA; Nepal Nutrition Intervention Project–Sarlahi, Lalitpur, Nepal; Department of International Health, Johns Hopkins Bloomberg School of Public Health,, Baltimore, MD, USA; Department of International Health, Johns Hopkins Bloomberg School of Public Health,, Baltimore, MD, USA

**Keywords:** low birthweight, survey, validation, multiple imputation, low- and middle-income country

## Abstract

**Background:**

The Global Nutrition Target of reducing low birthweight (LBW) by ≥30% between 2012 and 2025 has led to renewed interest in producing accurate, population-based, national LBW estimates. Low- and middle-income countries rely on household surveys for birthweight data. These data are frequently incomplete and exhibit strong “heaping.” Standard survey adjustment methods produce estimates with residual bias. The global database used to report against the LBW Global Nutrition Target adjusts survey data using a new MINORMIX (multiple imputation followed by normal mixture) approach: *1*) multiple imputation to address missing birthweights, followed by *2*) use of a 2-component normal mixture model to account for heaping of birthweights.

**Objectives:**

To evaluate the performance of the MINORMIX birthweight adjustment approach and alternative methods against gold-standard measured birthweights in rural Nepal.

**Methods:**

As part of a community-randomized trial in rural Nepal, we measured “gold-standard” birthweights at birth and returned 1–24 mo later to collect maternally reported birthweights using standard survey methods. We compared estimates of LBW from maternally reported data derived using: *1*) the new MINORMAX approach; *2*) the previously used Blanc–Wardlaw adjustment; or *3*) no adjustment for missingness or heaping against our gold standard. We also assessed the independent contribution of multiple imputation and curve fitting to LBW adjustment.

**Results:**

Our gold standard found 27.7% of newborns were LBW. The unadjusted LBW estimate based on maternal report with simulated missing birthweights was 14.5% (95% CI: 11.6, 18.0%). Application of the Blanc–Wardlaw adjustment increased the LBW estimate to 20.6%. The MINORMIX approach produced an estimate of 26.4% (95% CI: 23.5, 29.3%) LBW, closest to and with bounds encompassing the measured point estimate.

**Conclusions:**

In a rural Nepal validation dataset, the MINORMIX method generated a more accurate LBW estimate than the previously applied adjustment method. This supports the use of the MINORMIX method to produce estimates for tracking the LBW Global Nutrition Target.

## Introduction

Low birthweight (LBW) is closely associated with a higher risk of neonatal death as well as cognitive and developmental impairment and long-term health problems in adulthood (1–3). The term LBW (birthweight <2500 g) encompasses both newborns born preterm (birth <37 complete weeks of gestation) and those who are small for gestational age (birthweights <10th centile of a sex-specific birthweight-for-gestational-age reference population) but not necessarily preterm (4, 5). Although gestational age information is routinely documented in most high-income countries, this remains challenging in many low- and middle-income settings (6). As a result, LBW remains an important indicator of newborn health globally. In 2012, all WHO member states endorsed the Global Nutrition Target to reduce LBW by ≥30% between 2012 and 2025, renewing interest in producing population-based LBW estimates at the country level. The joint WHO and UNICEF global database of LBW estimates tracks progress towards the target. Whereas crude survey data in low- and middle-income countries (LMIC) underestimate LBW prevalence, this database uses the recently developed MINORMIX method to better adjust survey-derived estimates to reflect the true prevalence of LBW (J Krasevec et al., unpublished results, 2020).

Population-based household surveys, like the Demographic and Health Surveys (DHS) and the Multiple Indicator Cluster Surveys (MICS), are the primary source of birthweight data in most LMIC. These surveys capture birthweight data either through reviewing official records (e.g., birthcards) or, when not available, by maternal report (7). However, birthweight data are not available for nearly one-third of newborns globally, and as many as two-thirds of newborns in Western Africa (8, 9). This is because many births still occur at home (8–10) and newborns delivered in facilities might not be consistently weighed or have incomplete records (7). Mothers delivering in facilities and their offspring's health might not be representative of the whole population, which can bias estimates when they are overrepresented in the sample of birthweights (2). Even mothers of newborns weighed at birth can be unable to produce a birthweight record or recall birthweight accurately at the time of the survey, which could be administered ≤5 y after a birth (7, 11–14). As a result, birthweight data are often missing, inaccurate, or imprecise, and often “heaped” or rounded to multiples of 100 or 500 g.

Using birthweight data from 88 DHS datasets, Blanc and Wardlaw (15) developed a method to adjust survey-based LBW estimates to account for heaping and missing birthweights, based on perceived birth size. This correction was used to adjust household survey-based LBW estimates in the UNICEF global database starting in 2004 (2). However, there has been growing concern around residual bias in LBW estimates generated using this approach.

The Lancet Low Birthweight Investigator Group, comprised of members from UNICEF, WHO, the London School of Hygiene and Tropical Medicine, and the Johns Hopkins Bloomberg School of Public Health, recently developed a new approach to adjust estimates of LBW calculated from household survey data for use in the global database to track the LBW Global Nutrition Target (16). Extending previous research describing the distribution of birthweight derived from a variety of large datasets, this group developed an approach involving 3 steps (16). First, the quality of survey datasets is evaluated against a set of criteria; only surveys meeting the quality criteria are used to estimate LBW. Second, multiple imputation (MI) is used to impute missing birthweights using covariates available in the dataset. Third, to address the problem of heaping of birthweights, a mixture model comprising 2 normal distributions is fitted to the data from which the proportion of births with a birthweight <2500 g can be estimated. The Working Group explored fitting 1-, 2-, and 3-component normal mixture models using >200 DHS datasets and identified the 2-component normal mixture model as the preferred method for generating LBW estimates (16). The MI followed by normal mixture model approach has been abbreviated to MINORMIX. The approach was developed using high-quality birthweight data from the United States. However, the method has not been validated in an LMIC population due to the lack of a gold-standard comparator.

In this article, we evaluate the MINORMIX approach described above using a household survey dataset of maternal reports of birthweight against that of gold-standard paired measured birthweights collected as part of a large community-randomized trial in rural Nepal. We also assess the performance of the MINORMIX method relative to alternative LBW estimation methods, including *1*) the crude unadjusted estimate as would be reported in a DHS report, and *2*) the Blanc and Wardlaw method used in the previous global database, to gauge whether the MINORMIX approach offers a substantial improvement over existing methods. Finally, we assess the relative contribution of the 2 components of the MINORMIX method to the overall performance of the approach, specifically *1*) MI of missing birthweights, and *2*) fitting a normal model to account for heaping.

## Methods

### Study setting

The study was carried out in the rural district of Sarlahi, Nepal. This district is in the terai region (plains) along the border with Bihar, India. A little more than one-third of women aged ≥15 y can read and write (17). Over one-third of its predominantly Hindu residents are aged <15 y, and ∼15% of married women reported having been younger than 15 y at the time of their first marriage (17).

### Ethical approval

The parent trial and the substudy both received ethical approval from the Johns Hopkins Bloomberg School of Public Health Institutional Review Board, Baltimore, MD, USA. Local approval was received from the Tribhuvan University Institute of Medicine, Kathmandu, Nepal, for the parent trial and from the Nepal Health Research Council, Kathmandu, Nepal, for the substudy.

### Parent trial

A randomized community-based trial, conducted from November 2010 to January 2017, examined the impact of the use of sunflower seed oil in full-body newborn massage on neonatal deaths and infections (registered at clinicaltrials.gov as NCT01177111). Locally resident female project workers visited married women aged 15–35 y at home every 5 wk to identify new pregnancies; pregnancies in women outside this age range were identified informally. Women who consented to participate were followed through delivery. Study staff visited as soon as possible after delivery, typically within 48 h, but visits conducted ≤72 h after birth were included in our substudy. At the first visit after birth, workers recorded the median of 3 measures of the baby's weight using a digital scale graduated to 10 g (Tanita BD-585) in addition to other data on the circumstances of the birth. These data serve as our gold-standard measure of each study subject's true birthweight. The date, time of birth, and weight of the newborn were also provided to the mother/caretaker on a small card.

### Substudy

We selected mother/child pairs from the parent trial for 1 additional follow-up visit with the aim to interview roughly the same numbers of mothers at each 1, 3, 6, 9, 12, 18, or 24 mo after birth. Mother/child pairs were excluded from this follow-up interview if they were part of a nonsingleton birth. Surveyed pairs were subsequently dropped from the dataset when simulating missing birthweights if *1*) weight data were collected >72 h after birth, *2*) covariates required for the analysis were missing, or *3*) the mother had previously been included in the study for another birth. Pairs were excluded from the final analysis if measured birthweight data were unavailable. Study staff requested participation in the homes of selected mothers, administered oral consent in Nepali or Maithili (a local language), and obtained a signature or thumbprint for those who agreed to participate. Specific to this analysis, mothers were asked standard questions from the DHS or MICS including birthweight, perceived birth size (categorized as very large, larger than average, average, smaller than average, and very small), and whether they had a written record of their child's birthweight. If mothers reported a written record of the birthweight, outside of the record provided as part of the trial (e.g., hospital birth record), the recorded birthweight was captured in line with the approach used in national surveys. These birth record and maternally recalled data serve as our measure of birthweight collected using standard household survey methods and are referred to as “maternally reported” birthweight data throughout the article.

### Household survey birthweight data and simulated missingness

In the 2011 Nepal DHS, 67.3% of birthweights were missing for rural households. However, in our substudy, 95.1% of mothers reported a birthweight. Because the objective of our study was to assess the validity of the MINORMIX approach in a typical LMIC survey dataset, we aimed to produce a survey dataset that simulated the missingness commonly observed in national household surveys. We developed an approach to simulate missingness in our trial dataset reflecting the patterns of missingness observed in the 2011 Nepal DHS dataset.

First, we assessed the covariates associated with missing birthweight data in the 2011 Nepal DHS. We hypothesized a priori that missing birthweight in the 2011 Nepal DHS may be associated with whether the mother had ≥4 antenatal care (ANC) visits, birth order of the child, birth size, child sex, singleton/multiple birth, maternal height, maternal BMI, maternal smoking status, birth interval, maternal education, maternal age, and household wealth. We restricted the 2011 Nepal DHS dataset to only households in rural areas to ensure comparability with our study population. We reconstructed the wealth quintile variable to reflect the distribution of wealth in rural households. We then conducted a logistic regression using individual sampling weights to account for differential probability in selection to investigate the association of missing birthweight with the above variables (17, 18). **Supplemental Table 1** presents the odds of missing birthweight by the 12 a priori selected characteristics of the mother or birth, both individually and adjusting for the other variables. The odds of missing birthweight were associated with birth size, single compared with multiple births, parity, having ≥4 ANC visits, wealth quintile, and maternal education. However, single compared with multiple birth was not included when adjusting the study dataset because this was an exclusion criterion in our substudy.

Based on the results of the patterns of missingness in rural households from the 2011 Nepal DHS dataset, we removed birthweights in the trial dataset to simulate the missingness in the Nepal 2011 DHS. We simulated the missingness in our dataset by applying the associations observed in the 2011 Nepal DHS to calculate the predicted probability of each respondent in the trial dataset not reporting a birthweight based on their characteristics and characteristics of the birth. Using computer-generated random numbers and the predicted probability of missingness, ∼63% of maternally reported birthweights were then set to missing. Accounting for the initial 4.9% missing birthweights, we generated a dataset of reported birthweights similar in missingness to the 2011 Nepal DHS. The resulting patterns of birthweight missingness for the 5 associated variables in the simulated dataset compared with the Nepal 2011 DHS are presented in **Supplemental Table 2**. This dataset with simulated missing birthweights was used for the primary analysis of performance of the LBW adjustment methods.

### Birthweight data quality assessment

All datasets included in the UNICEF/WHO LBW database must meet 3 criteria for minimum level of data quality (16). These criteria include: *1*) a minimum sample size of ≥200 birthweights available in the dataset, *2*) a minimum of ≥30% of births having a birthweight, and *3*) no indication of an implausible distribution and/or severe heaping of the birthweights defined as *a*) ≤55% of all birthweights falling on the 3 most frequent birthweights, *b*) ≤10% of all birthweights weighing ≥4500 g, and *c*) ≤5% of births on tail ends of 500 g and 5000 g. We assessed the maternally reported and simulated missing birthweight data quality against the UNICEF/WHO LBW database inclusion criteria to gauge the comparability of the data used in the validation exercise (16).

### Methods for estimating LBW using household survey data

We applied the MINORMIX approach, a 2-component normal mixture model fit to a dataset with multiply imputed missing birthweight, to the maternally reported dataset with simulated missing birthweights to estimate LBW. We also estimated LBW from the maternally reported dataset with simulated missing birthweights using existing methods, including *1*) a crude, unadjusted proportion, and *2*) the Blanc–Wardlaw method. We compared the LBW estimates generated using each method with those from our dataset of measured (gold-standard) birthweights.

The MINORMIX approach applies 2 steps. First, an MI procedure is used to impute missing birthweights in the dataset. The MI was conducted with 5 repetitions using the variables associated with birthweight identified by the Working Group regression analysis of 88 post-2000 DHS datasets (16). These variables included: perceived birth size, sex of the child, maternal height, maternal BMI, and parity; singleton/multiple birth was not used because being a multiple birth was an exclusion criterion for our substudy. Second, a 2-component normal mixture model is fit to the dataset inclusive of imputed birthweights. The distribution of birthweights is assumed to be composed of 2 subpopulations: *1*) a primary normal distribution that accounts for most birthweights, and *2*) a secondary normal distribution that captures the smallest newborns in the left tail of the distribution (19). Combining these 2 curves, the area under the overall function with a cut point at 2500 g equals the proportion of LBW newborns.

Common alternative methods for estimating LBW include presenting unadjusted estimates and application of the Blanc–Wardlaw method. Currently, DHS reports include LBW estimates based on a crude, unadjusted estimate of the proportion of birthweights <2500 g. The previous global database for tracking LBW used the Blanc–Wardlaw approach to adjust survey-derived birthweight data. The Blanc–Wardlaw approach is a 2-step adjustment procedure (15). First, to account for heaping of birthweights on 100- or 500-g increments, the Blanc–Wardlaw approach reclassifies 25% of births reported as exactly 2500 g as LBW. Second, to account for missing birthweights, babies without reported birthweight data are classified as low or normal birthweight based on reported birth size and the distribution of LBW within each perceived birth size category. For example, in a given survey, if 60% of children reported to be “very small” had a reported birthweight <2500 g, then 60% of children missing birthweight data and reported to be “very small” would be classified as LBW.

We further assessed the relative contribution of MI (to account for missing birthweights) and curve fitting (to address heaping) on the accuracy of LBW estimates produced through the MINORMIX method. We compared the estimates of LBW generated using MI alone by calculating LBW with no heaping adjustment following the MI of missing birthweights. We also compared the heaping adjustment alone by fitting 1- and 2-component normal curves to the simulated missing dataset without imputing missing birthweights prior to estimation. All analyses were performed using Stata version 14.0 (StataCorp).

## Results

**FIGURE 1:**
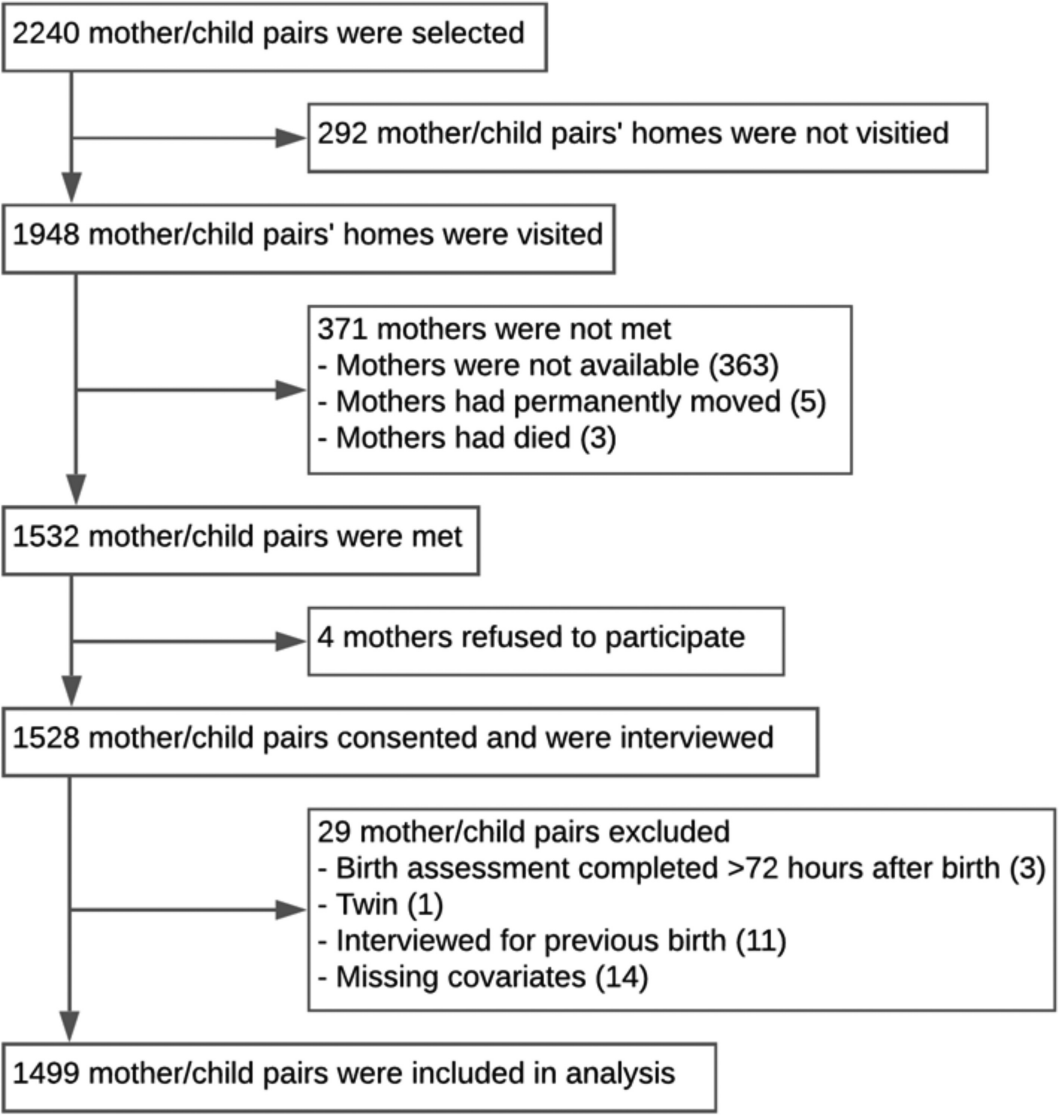
Flowchart for participant selection.

In total, 1528 mothers consented to participate and were interviewed as part of the substudy (**Figure 1**). Twenty-nine participants were excluded—birth assessment >72 h after birth (*n* = 3), twin delivery improperly included in the substudy (*n* = 1), repeat participation due to inclusion of >1 child per mother (*n* = 11), or missing covariates (*n* = 14). The final dataset for generating the dataset with simulated missing birthweights included 1499 mother/child pairs. Only 74 (4.9%) children were missing maternally reported birthweights, where the mother reported the child was not weighed (*n* = 21), was uncertain if the child was weighed (*n* = 6), or was weighed but could not provide a numerical weight (*n* = 47). Of the 1499 mothers who were asked if they had a card with a birthweight record, only 22 (1.5%) presented cards provided by a facility. To mimic circumstances of a DHS or MICS data collection, we used birthweights recorded on these facility cards in our dataset of maternally reported birthweights. Of the 1499 pairs included in the simulated missingness analysis, 16 (1.1%) children were missing a gold-standard digital weight measurement within 72 h of birth for reasons including death before measurement (*n* = 14), parental refusal of weight measurement (*n* = 1), and missing weight measurement (*n* = 1). These children were excluded from the comparison of LBW estimation methods.

**FIGURE 2:**
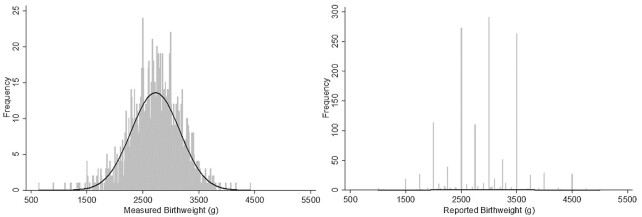
Measured and reported birthweights (*n* = 1483) with fitted normal curve.

The mean measured birthweight was 2724.3 ± 433.9 g for newborns weighed within 72 h of birth. Based on measured weight, 27.7% had birthweights <2500 g. Birthweight based on maternal report 1–24 mo after birth was higher on average, with a mean of 2884.6 ± 607.0 g and only 17.1% (95% CI: 15.2, 19.2%) of newborns classified as LBW. **Figure 2** displays a histogram of the measured and reported birthweight datasets. The measured birthweights appear to be generally normally distributed with a left tail that diverges somewhat from the normal curve. Strong heaping is evident in the histogram of reported birthweights with 71.4% of all birthweights being multiples of 500 g, of which 19.1% were exactly 2500 g (**Table 1**). Applying the Working Group's birthweight data quality criteria to the simulated missing dataset confirmed substantial heaping because 60.6% of the reported birthweights fell on 2500, 3000, and 3500 g. Heaping was still evident in the MI dataset, although significantly reduced compared with either the maternally reported or simulated missing dataset.

**TABLE 1:** Assessment of household survey birthweight data quality^1^

	Reported	Reported with simulated missingness	Reported with simulated missingness and multiple imputations
UNICEF LBW database quality criteria			
*1*) Total number of births	1499	1483	1483
*2*) Percentage of births with a birthweight	95.1	32.0	100
*3*) Heaping criteria			
*a*) Percentage of all birthweights falling on the 3 most frequent birthweights	58.0	60.6	19.6
*b*) Percentage of birthweights ≥4500 g	2.1	1.3	0.8
*c*) Percentage of birthweights on tail ends of 500 g and 5000 g	0.1	0.0	0.0
Additional quality indicators			
Percentage of birthweights weighing exactly 2500 g	19.1	18.7	6.1
Percentage on 500s	71.4	71.6	23.1

1 LBW, low birthweight.

**FIGURE 3:**
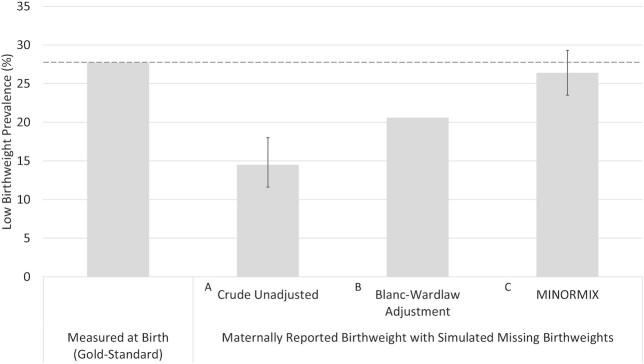
Comparison of low birthweight estimates generated from gold-standard measured birthweight compared with 3 methods for adjusting reported birthweight dataset with simulated missingness, including (A) no adjustment reflecting the reported value in a DHS, (B) the Blanc–Wardlaw method as applied in the previous global database, and (C) the MINORMIX method used for the current global database released in 2019 (16). DHS, Demographic and Health Survey; MINORMIX, multiple imputation followed by normal mixture.

**Figure 3** compares LBW estimates generated using MINORMIX, the Blanc–Wardlaw approach, and crude estimate applied to the simulated missing birthweight dataset against the measured gold-standard data. The crude (unadjusted) LBW prevalence, meant to mimic estimates presented in a DHS report, was nearly 50% lower than the measured gold-standard estimate of 27.7%. The estimated proportion LBW after applying the Blanc–Wardlaw method was 20.6%, which captured only three-quarters of the true LBW prevalence. The MINORMIX LBW estimate of 26.4% (95% CI: 23.5, 29.3%) was notably closer to the measured LBW value, and the confidence bounds encompassed the measured LBW point estimate of 27.7%.

We compared the estimates of LBW generated through MI and curve-fitting, independently and jointly (**Table 2**). Whereas each of the adjustment methods brought the LBW estimate closer to our true measured LBW proportion of 27.7%, combining the MI and heaping adjustment methods resulted in the closest estimates to our gold standard. Adjusting for heaping without MI, fitting a single normal curve and a 2-component mixture model increased the LBW estimate to 24.0% (95% CI: 20.9, 27.1%) and 25.6% (95% CI: 22.4, 28.8%), respectively. MI alone increased the LBW estimate to 23.6% (95% CI: 20.0, 27.0%). MI and fitting a 1-component normal curve estimated LBW at 27.1% (95% CI: 23.1, 31.2%), slightly closer to the gold-standard estimate than the MINORMIX approach. The 2-component mixture model was unable to identify 2 separate subpopulations when fit to both the simulated missing and some of the MI datasets as evidenced by the small variance around the second component of these mixture models (**Supplemental Table 3**).

**TABLE 2:** Low birthweight point estimates (%) calculated using 3 methods to adjust for heaping on reported birthweight data with simulated missingness (*n* = 1483) with and without imputation for missing birthweights

	Adjustment for heaping:		
	No adjustment for heaping	One-component normal curve	Two-component normal mixture model
Adjustment for missing birthweight:	% (95% CI)	% (95% CI)	% (95% CI)
No imputation for missing birthweights	14.5^1^ (11.6, 18.0)	24.0^2^ (20.9, 27.1)	25.6^2^ (22.4, 28.8)
Missing birthweights imputed with multiple imputation (*r* = 5)	23.5^3^ (20.0, 27.0)	27.1^4^ (23.1, 31.2)	26.4^4^ (23.5, 29.3)

1 No adjustment for heaping or missing birthweight.

2 Adjusted for heaping, no adjustment for missing birthweight.

3 Adjusted for missing birthweights, no adjustment for heaping.

4 Adjusted for missing birthweights and heaping.

## Discussion

Existing methods to account for heaping and missing birthweight data from household surveys are insufficient and result in underestimation of the prevalence of LBW, leading to inaccurate information for global monitoring and program planning. To address biased LBW estimates, the Lancet Low Birthweight Investigator Group proposed MI of missing birthweights and fitting a 2-component normal curve to birthweight data (MINORMIX) as the most accurate and parsimonious method to adjust LBW estimates derived from household survey data (16). To validate the MINORMIX approach in an LMIC population, we compared estimates of LBW calculated by applying the MINORMIX approach to a household survey dataset against gold-standard measured birthweight in rural Nepal. Our dataset was unique because it contained paired gold-standard measured birthweight and maternally reported birthweights for individual children, allowing for a direct comparison within the study population. In our birthweight dataset, which exhibited very strong heaping and relied almost exclusively on maternal reports rather than birth cards, the MINORMIX approach produced valid estimates of LBW in the study population, generating an estimate of LBW that encompassed the gold-standard measured proportion LBW. It also produced more accurate LBW estimates than previous methods, including crude estimates and the Blanc–Wardlaw method.

The crude LBW estimates currently reported by major household survey programs (e.g., DHS) are likely biased in that they do not account for missing birthweights, nor do they adjust for birthweight heaping. Using our dataset of reported birthweights simulated to represent patterns of missingness in the 2011 Nepal DHS, the crude LBW estimate of 14.5% (95% CI: 11.6, 18.0%) captured barely half the true proportion of LBW based on the gold-standard measured LBW of 27.7% in the same group of births. The crude LBW estimate was similar to those reported in the rural population in the 2011 (12.5%) and 2016 (12.9%) Nepal DHS reports (17, 20). With many country survey reports likely underestimating LBW prevalence by not accounting for biases, policies and programs might not be commensurate with the issue's true magnitude. A recent application of the MINORMIX method to 226 survey datasets in 86 LMIC estimated that the average LBW prevalence was ∼35% greater than unadjusted survey estimates (J Krasevec et al., unpublished results, 2020). Our analysis results suggest that the new method does a better job of estimating the true prevalence of LBW than the previous Blanc–Wardlaw method and supports the findings associated with its application.

The previous methods to adjust LBW estimates in the global database (2), as developed by Blanc and Wardlaw (15), might not entirely correct for biased reporting of LBW in surveys. In this analysis, the Blanc–Wardlaw adjusted estimate was 25% lower than the measured LBW prevalence. The Blanc–Wardlaw approach applies an adjustment for heaped birthweights based on an averaged pattern of reported birthweights (15). However, heaping can be highly variable, and the resulting Blanc–Wardlaw-adjusted LBW estimate is particularly sensitive to this variation (21). Additionally, the method relies on a consistent perception of birth size within a study population. Studies have assessed the relation of birthweight and perceived birth size within DHS datasets and found that mean birthweight generally decreased with decreasing birth size, consistent with our findings (results not shown here) (11, 12, 14, 22). However, mothers' perception of birth size can be affected by various neighborhood and regional factors specific to a setting that shape a reference for how mothers assess their child's size (23).

We explored the relative contribution of both adjusting for missing birthweights and adjusting for heaping through the MINORMIX approach. We found that the MI alone brought the LBW estimate closer to the gold standard. Fitting a normal mixture model alone also yielded an estimate closer to the gold-standard estimate. However, combining MI and fitting a normal mixture model yielded point estimates closest to the real value with good precision, supporting the MINORMIX method employed in the current global database (8, 16).

Wilcox and colleagues described birthweight as having a Gaussian distribution comprised of 2 subpopulations: a “predominant” subpopulation with a Gaussian distribution that encompasses most birthweights, and a “residual” subpopulation made up primarily of LBW newborns (24, 25). Other analyses have found that birthweight data can be fitted using normal mixture models and the number of components may vary (19, 26). The 2-component mixture model had trouble identifying subpopulations in our study, as evidenced by the small variance around the mixture model's second component. The 1-component normal curve point estimate was slightly closer to the gold-standard estimate. This might have been due at least in part to the inclusion of only singleton births in our study and the exclusion of 14 children who died before birthweights were obtained. Both multiple births and early neonatal deaths are more likely to have LBWs (27, 28). Their exclusion from this study might mean that the left tail of the distribution in our study population is less skewed than would exist in a population representative of all births, contributing to the inability to distinguish 2 populations in the reported birth dataset.

Our study used accurate and calibrated scales of research quality to minimize measurement error and produced a similar measured birthweight distribution pattern observed in prior high-quality studies (19, 24, 26). A limitation of using these measurements as our gold standard is that newborns were weighed ≤72 h after birth. In these first hours of life, newborns generally lose weight before growth and weight gain are observed. However, we intended to validate these methods rather than provide an estimate of prevalence. Our study population also had a relatively high proportion of LBW newborns, and we excluded multiple births and early neonatal deaths, tempering the generalizability of our validation of these methods.

A much higher proportion of mothers in our study reported a birthweight at follow-up than seen in standard household surveys in Nepal. In the case of home births, mothers likely recalled the birthweight measured during the parent trial because this would have been the only birthweight provided to them. Children delivered in a facility might have been weighed at the facility and during the parent trial participation. In the latter case, we assumed the mother was reporting the weight measurement provided to them during the parent trial. It is not uncommon for a high proportion of birthweights to be missing in large household surveys (9). We removed a relatively high percentage of birthweights to mimic the missingness patterns in rural households in the 2011 Nepal DHS dataset. Birthweights were removed based on observed patterns of missingness, which included covariates also included the MI approach, ensuring the missing at random assumption underlying the MI approach was met (29) and that our dataset closely resembled the birthweight data that would be collected through a standard household survey.

Additionally, considering that most birthweights in our dataset were recalled by mothers rather than transcribed from birth cards, which resulted in strong heaping, our dataset might represent a relatively extreme case of birthweights that would require adjustment. Although strong heaping was evident in the maternally reported birthweight dataset, we removed >60% of birthweights to simulate missingness in the Nepal 2011 DHS. MI was unlikely to impute missing values on multiples of 100 or 500 g, resulting in an approximately two-thirds reduction in indicators of heaping severity compared with the near-complete maternal dataset. As a result, removing heaped birthweights prior to imputation and curve-fitting might have improved the curve fitting approach's performance. Assessment of these methods using other validation datasets would improve our understanding of their performance in populations with different birthweight distributions.

In conclusion, LBW estimates in survey reports, and the previous methods employed in the global database to adjust LBW estimates to address heaping and missing birthweights, result in underreporting of LBW. Our validation exercise suggests the new MINORMIX method employed in the current global database developed by the Lancet Low Birthweight Investigator Group produces more accurate LBW estimates. The 2-component normal mixture model with MI method generated LBW estimates more accurate than the Blanc–Wardlaw method when applied to this rural Nepal validation dataset with high LBW prevalence, a large degree of heaped birthweights derived primarily from maternal recall, and a high proportion of missing birthweights. Although the MINORMIX approach was developed using high-quality data from the United States, the method performs significantly better than alternatives when applied as intended to maternally reported birthweight data collected through household surveys in an LMIC. Applying these methods to adjust for biased birthweight estimates can be more complex than the presentation of crude estimates based on those with reported birthweights only. However, given the significant burden of LBW on population health, the consistent production of more accurate LBW estimates for stakeholders warrants the increased complexity. This validation exercise supports the use of the MINORMIX approach to produce more accurate estimates for reporting against the LBW Global Nutrition Target and ensure valid data for global monitoring and response.

## Supplementary Material

nxab417_Supplemental_File

## Data Availability

Data described in the manuscript, code book, and analytic code will be made available upon request pending application and approval of the principal investigator.

## Notes

This work was supported by the Improving Coverage Measurement grant (OPP1084442) from the Bill & Melinda Gates Foundation, the Nepal Oil Massage Study grant (OPP1084399) from the Bill & Melinda Gates Foundation, and a grant (R01HD060712) from the National Institutes of Health. The funders had no role in the design of the study and collection, analysis, and interpretation of data or in writing the manuscript.

Author disclosures: The authors report no conflicts of interest.

Supplemental Tables 1–3 are available from the “Supplementary data” link in the online posting of the article and from the same link in the online table of contents at https://academic.oup.com/jn/.

Abbreviations used: ANC, antenatal care; DHS, Demographic and Health Survey; LBW, low birthweight; LMICs, low- and middle-income countries; MI, multiple imputation; MICS, Multiple Indicator Cluster Surveys; MINORMIX, multiple imputation followed by normal mixture.
