# Supplementary material for: Validation of MINORMIX Approach for Estimation of Low Birthweight Prevalence Using a Rural Nepal Dataset
Source: J Nutr. 2021 Dec 9;152(3):872–9. doi: 10.1093/jn/nxab417 (PMC8891178; doi:10.1093/jn/nxab417)
Supplement: nxab417_Supplemental_File [file nxab417_supplemental_file.docx]

**Supplemental Table 1: Unadjusted and adjusted odds ratios (OR) for missing birthweight by socioeconomic and health risk factors among rural households in the 2011 Nepal DHS survey (n=4215)**

|  | **n** | **Unadjusted OR  (95% CI)** | **p-value** | **n** | **Adjusted OR  (95% CI)** | **p-value** |
| --- | --- | --- | --- | --- | --- | --- |
| **Birth size** |  |  | 0.006 |  |  | 0.002 |
| Very large (ref) | 80 | - |  | 43 | - |  |
| Larger than average | 686 | 0.89 (0.49, 1.60) |  | 334 | 2.71 (0.93, 7.87) |  |
| Average | 2666 | 1.18 (0.65, 2.15) |  | 1250 | 3.96 (1.43, 10.94) |  |
| Smaller than average | 601 | 1.82 (0.97, 3.39) |  | 308 | 6.06 (2.03, 18.09) |  |
| Very small | 178 | 1.06 (0.54, 2.10) |  | 93 | 2.25 (0.89, 5.65) |  |
| **Child sex** |  |  | 0.831 |  |  | 0.343 |
| Male (ref) | 2191 | - |  | 1043 | - |  |
| Female | 2024 | 1.02 (0.87, 1.18) |  | 985 | 0.90 (0.72, 1.12) |  |
| **Single vs Multiple** |  |  | 0.055 |  |  | 0.029 |
| Singleton (ref) | 4159 | - |  | 2000 | - |  |
| Multiple births | 56 | 0.45 (0.20, 1.02) |  | 28 | 0.29 (0.09, 0.88) |  |
| **Maternal height (cm)** | 2034 | 0.96 (0.94, 0.99) | 0.007 | 2028 | 0.99 (0.95, 1.02) | 0.378 |
| **Maternal BMI (kg/m2)** | 2033 | 0.92 (0.88, 0.96) | <0.001 | 2028 | 1.01 (0.95, 1.06) | 0.842 |
| **Maternal smoking status** |  |  | <0.001 |  |  | 0.441 |
| Nonsmoker (ref) | 3483 | - |  | 1677 | - |  |
| Smoker | 732 | 4.08 (2.82, 5.90) |  | 351 | 1.22 (0.73, 2.04) |  |
| **Parity** |  |  | <0.001 |  |  | <0.001 |
| Primiparous (ref) | 1314 | - |  | 671 | - |  |
| Second or third child | 1842 | 2.42 (1.99, 2.93) |  | 869 | 2.09 (1.34, 3.24) |  |
| Fourth child or greater | 1059 | 6.79 (5.02, 9.19) |  | 488 | 3.38 (1.98, 5.77) |  |
| **ANC status** |  |  | <0.001 |  |  | <0.001 |
| Less than 4 visits (ref) | 1646 | - |  | 811 | - |  |
| 4 visits or more | 1536 | 0.16 (0.12, 0.21) |  | 727 | 0.29 (0.22, 0.39) |  |
| Not recorded | 1033 | 0.76 (0.59, 0.98) |  | 490 | 1.23 (0.88, 1.71) |  |
| **Rural wealth quintile** |  |  | <0.001 |  |  | <0.001 |
| WQ 1 (ref) | 1436 | - |  | 668 | - |  |
| WQ 2 | 976 | 0.47 (0.34, 0.64) |  | 471 | 0.52 (0.31, 0.87) |  |
| WQ 3 | 752 | 0.28 (0.20, 0.39) |  | 389 | 0.36 (0.21, 0.61) |  |
| WQ 4 | 579 | 0.16 (0.11, 0.22) |  | 291 | 0.23 (0.14, 0.38) |  |
| WQ 5 | 472 | 0.06 (0.04, 0.08) |  | 209 | 0.11 (0.06, 0.19) |  |
| **Birth interval (mos)** | 4212 | 1.01 (1.01, 1.01) | <0.001 | 2028 | 1.00 (0.99, 1.01) | 0.791 |
| **Maternal education** |  |  | <0.001 |  |  | 0.027 |
| none (ref) | 2157 | - |  | 1045 | - |  |
| any primary | 864 | 0.53 (0.39, 0.72) |  | 413 | 1.16 (0.76, 1.76) |  |
| any secondary or higher | 1194 | 0.15 (0.11, 0.20) |  | 570 | 0.65 (0.44, 0.96) |  |
| **Maternal age (yrs)** |  |  | <0.001 |  |  | 0.216 |
| 15-19 | 850 | - |  | 426 | - |  |
| 20-34 | 3032 | 1.44 (1.19, 1.74) |  | 1444 | 1.26 (0.88, 1.82) |  |
| 35 and older | 329 | 3.02 (1.98, 4.59) |  | 158 | 0.71 (0.33, 1.50) |  |

**Supplemental Table 2: Missing birthweight for each significantly associated variable among rural households in the 2011 Nepal DHS dataset**

|  | **Nepal 2011 DHS** | **Simulated Missingness in Trial Dataset** |
| --- | --- | --- |
|  | **% missing** | **% missing** |
| **Birth size** | n=4215 | n=1483 |
|  |  |  |
| Very large | 63.7 | 75.0 |
| Larger than average | 60.9 | 60.8 |
| Average | 67.5 | 67.6 |
| Smaller than average | 76.1 | 78.1 |
| Very small | 65.1 | 76.7 |
| **Parity** |  |  |
| Primiparous | 49.6 | 58.8 |
| Second or third child | 70.4 | 67.3 |
| Fourth child or greater | 87.0 | 69.0 |
| **ANC status** |  |  |
| Less than 4 visits | 82.6 | 72.3 |
| 4 visits or more | 43.4 | 53.8 |
| Not reported | 78.3 | 74.6 |
| **Rural wealth quintile** |  |  |
| WQ 1 | 88.6 | 78.2 |
| WQ 2 | 78.5 | 72.9 |
| WQ 3 | 68.3 | 69.2 |
| WQ 4 | 55.1 | 62.9 |
| WQ 5 | 30.4 | 45.0 |
| **Maternal education** |  |  |
| none | 82.2 | 73.1 |
| any primary | 70.8 | 65.9 |
| any secondary or higher | 40.9 | 53.2 |
| **Total** | 67.3 | 68.0 |

**Supplemental Table 3: One and two component normal models fit to gold-standard measured birthweights, reported birthweight data with simulated missingness, and first imputation on simulated missing dataset (n=1483).**

|  | **Mean (SD) (g)** | **Proportion of Population in Each Component** |
| --- | --- | --- |
| **Measured** |  |  |
| Single normal curve | 2724 (434) | - |
| Two-component normal mixture |  |  |
| Normal component 1 | 2512 (634) | 0.141 |
| Normal component 2 | 2759 (380) | 0.859 |
| **Simulated Missing** |  |  |
| Single normal curve | 2916 (568) | - |
| Two-component normal mixture |  |  |
| Normal component 1 | 2756 (568) | 0.785 |
| Normal component 2 | 3500 (5.1E-71) | 0.215 |
| **Simulated Missing - First Imputation** |  |  |
| Single normal curve | 2898 (577) | - |
| Two-component normal mixture |  |  |
| Normal component 1 | 2854 (574) | 0.931 |
| Normal component 2 | 3500 (6.0E-15) | 0.069 |

SD standard deviation
